# Controversies in treating nutcracker syndrome

**DOI:** 10.1186/s42155-025-00544-z

**Published:** 2025-03-28

**Authors:** Vitorio Perić, Thomas Ferenc, Tomica Bratić, Jana Bebek, Ivan Antun Mašić, Filip Ferega, Vid Vrčić, Danko Milošević, Helga Sertić Milić, Vinko Vidjak

**Affiliations:** 1 Department of Diagnostic and Interventional Radiology, University Hospital Merkur, Zagreb, Croatia; 2 Department of Diagnostic and Interventional Radiology, University Hospital Dubrava, Zagreb, Croatia; 3 School of Medicine, University of Zagreb, Zagreb, Croatia; 4 Department of Pediatrics, Zabok General Hospital and Croatian Veterans Hospital, Zabok, Croatia

## Abstract

Nutcracker syndrome (NCS) is a relatively uncommon vascular condition characterized by compression of the left renal vein (LRV), resulting in a variable spectrum of nonspecific symptoms, including hematuria, flank pain, varicocele, and pelvic congestion syndrome. NCS can be classified into anterior and posterior types regarding the origin of LRV compression: anterior NCS occurs when LRV is compressed between the aorta and superior mesenteric artery, whereas posterior NCS involves LRV compression between the aorta and the spine. Despite advancements in diagnostic modalities, including Doppler ultrasound, computed tomography, magnetic resonance imaging, and invasive techniques like phlebography, there is still no globally accepted diagnostic algorithm, leading to inconsistencies in diagnosis. Moreover, due to the lack of standardized treatment guidelines, the optimal management of anterior NCS remains a topic of debate. While conservative management is usually recommended in the pediatric population, invasive treatments—including surgical options like LRV transposition and renal autotransplantation, as well as interventional radiology procedures like stenting, present challenges such as stent migration, restenosis, and long-term material durability. Nevertheless, the emergence of 3D-printed stents offers potential improvements in patient-specific treatment, particularly in the pediatric population, yet their clinical efficacy and safety remain under investigation. This brief communication addresses the current discussions regarding anterior NCS management, emphasizing the need for standardized diagnostic algorithms, a multidisciplinary approach, and continued technological advancements to refine treatment possibilities and strategies. Further research is critical to resolve these controversies and establish a consensus on best practices.

## Introduction

Nutcracker syndrome (NCS) is a rare vascular disorder that involves the compression of the left renal vein (LRV), usually between the aorta and the superior mesenteric artery. It may lead to specific symptoms, including hematuria and flank pain, which can significantly affect a patient’s quality of life [1]. In male patients, a varicocele may be noted, whereas in females, pelvic congestion syndrome could be a possible manifestation [2].

Although NCS is a well-recognized medical condition, diagnosis and treatment remain debatable due to the broad spectrum of clinical manifestations, lack of standardized diagnostic criteria, and emerging innovative treatment modalities. One of the primary challenges in the management of NCS is the absence of a universally accepted diagnostic algorithm, resulting in variability of diagnostic criteria, leading to inconsistencies in patient evaluation. Another key controversy lies in determining the most appropriate treatment approach. While conservative management is generally preferred in the pediatric population, invasive treatments, including surgical and endovascular interventions, are considered for more severe cases. However, these interventions present challenges, including procedural risks, long-term complications, and uncertainty regarding material durability, particularly in younger patients. Emerging technologies, such as 3D-printed stents, hold promises for personalized treatment, yet their clinical application remains under investigation.

In terms of persistent uncertainties regarding NCS diagnosis and treatment, this brief communication aims to highlight and summarize the most important controversies in managing anterior NCS. The overview of current controversies underscores the necessity for standardized diagnostic guidelines, a multidisciplinary approach, and further research to optimize treatment strategies and improve patient outcomes.

## Diagnostics

Accurate diagnosis and assessment of NCS are crucial for determining the appropriate treatment strategy; however, the lack of globally recognized criteria complicates this process. Diverse imaging methods offer significant insights; however, each possesses distinct limits, and their reliability may vary among patient populations.

**Fig. 1:**
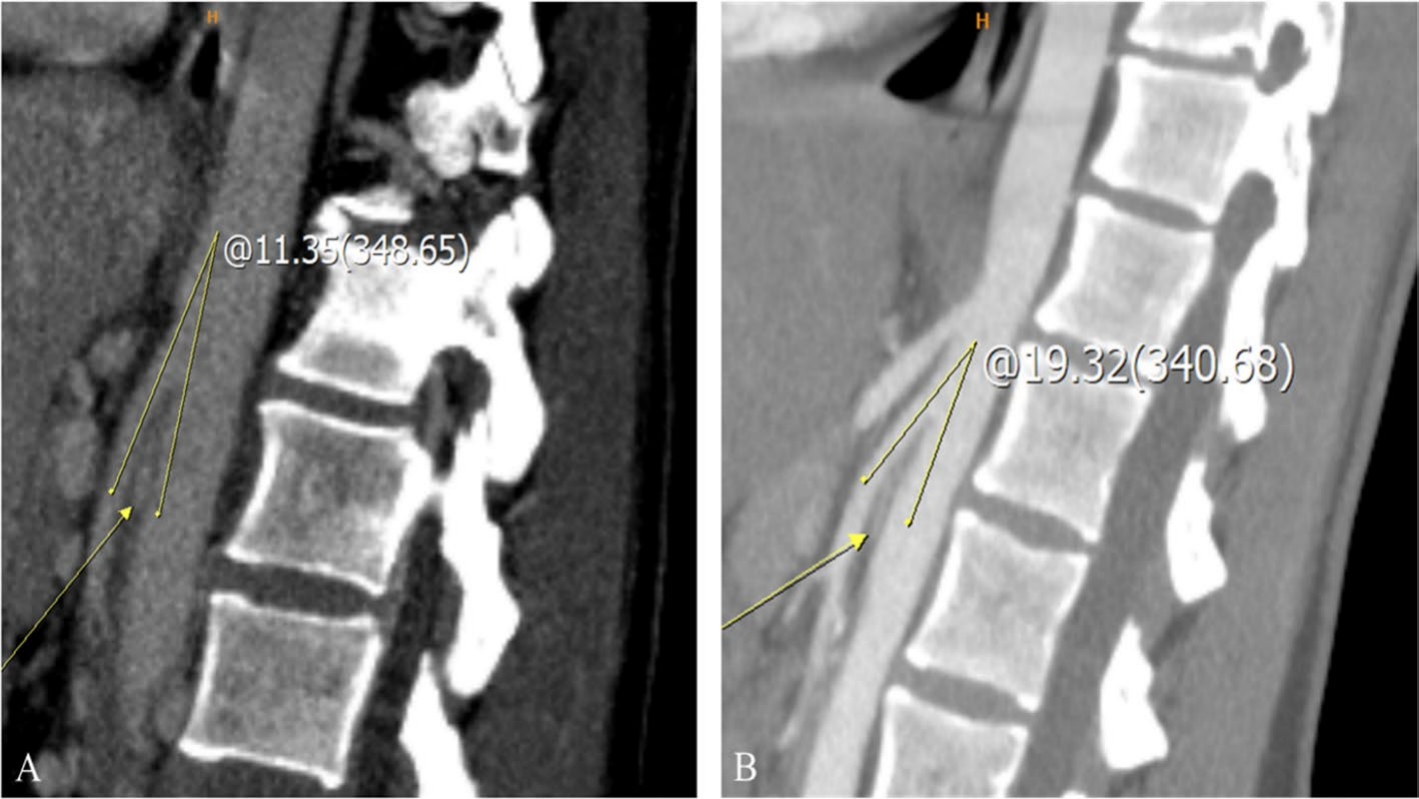
CT measurements of AMA in the sagittal plane: **A** portal venous phase—11° AMA indicative of NCS (yellow arrow depicting severely compressed LRV) incidentally detected in a 37-year-old female patient during staging for malignant disease; **B** arterial phase—19° AMA indicative of NCS (yellow arrow depicting severely compressed LRV) detected in a 17-year-old female patient who presented with a long-lasting non-specific abdominal pain

**Fig. 2:**
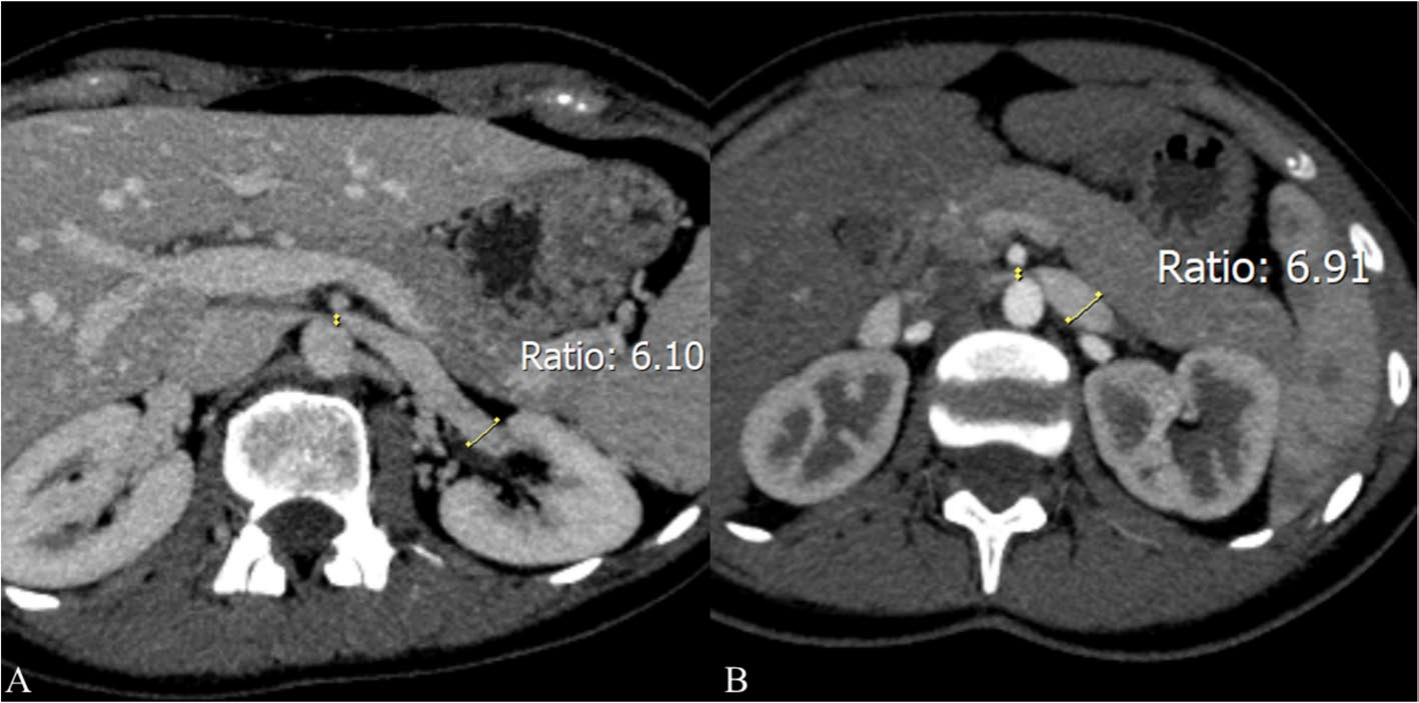
CT axial planes of the same patients with the ratio of the LRV diameter at the hilar and aorto-mesenteric level (the ratio > 5 indicates the presence of NCS): **A** portal venous phase – the ratio is 6.10; **B** arterial phase – the ratio is 6.91

Doppler ultrasound is a widely utilized non-invasive imaging modality that quantifies the peak flow velocity ratios between the compressed and non-compressed segments of the LRV. A peak systolic velocity ratio > 4.2–5.0 between the aortomesenteric segment and the hilar portion is a key diagnostic criterion for NCS [3]. Nonetheless, these cut-off values may lack reliability since they might be affected by factors such as patient hydration, respiratory fluctuations, and operator technique, resulting in diagnostic errors. Cross-sectional imaging, including computed tomography (CT) and magnetic resonance imaging (MRI), is frequently employed to assess the aortomesenteric angle (AMA) and the degree of compression in the left renal vein (LRV). An AMA of less than 41° has exhibited 100% sensitivity but only 55.6% specificity for NCS, highlighting the potential for false positive outcomes [4] (Fig. 1). Another commonly used CT criterion for NCS is a hilar to aortomesenteric LRV diameter ratio of ≥ 4.9, which exhibits excellent specificity (100%) but may not consistently identify milder cases. The "beak sign" shown on axial CT images is considered a suggestive finding; however, its interpretation may differ across radiologists, resulting in significant interobserver variability [5, 6] (Fig. 2). Certain studies propose a "compression ratio" of 2.25 as a diagnostic benchmark, with a sensitivity and specificity of 91% [2]. While this data may enhance the suspicion of NCS in symptomatic patients, anatomical variances and discrepancies in imaging techniques can influence their accuracy. Phlebography, when paired with pressure gradient monitoring, facilitates direct viewing and quantification of the pressure differential at the compression site for a more invasive diagnostic assessment. A pressure gradient of 3 mmHg is frequently utilized as a marker of substantial stenosis; however, this threshold is contentious, as variables including patient posture and hydration may affect outcomes. Furthermore, various organizations may employ slightly divergent measurement methodologies, complicating establishing a universally recognized standard.

## Treatment modalities

Several modalities are available for treating NCS, including conservative and invasive procedures (Table 1). Conservative management is frequently recommended for pediatric patients or those exhibiting mild symptoms, as it is believed that with growth and weight gain over time, the compression of the veins may be alleviated—approximately 75% of mild to moderate cases resolved symptoms after conservative therapy [7]. However, in symptomatic or severe cases, invasive intervention might be indicated.

**Table 1:** Summary table comparing treatment modalities

Treatment	Success Rate	Recurrence/Reintervention	Complications
LRV transposition	80% [9]	Rare but possible reintervention is needed [21]	Low
Renal Autotransplantation	Considered definitive treatment [12]	Rare recurrence	Surgical risks associated with kidney relocation
Endovascular Stenting	85.2% primary patency [22]	6.7% migration risk [17]	Restenosis, thrombosis, need for anticoagulation
3D-Printed Extravascular Stents	Promising results in small cohorts [15, 16]	Insufficient long-term data	Long-term safety not well established

*LRV* Left renal vein

### Surgical interventions: open and laparoscopic approaches

**Fig. 3:**
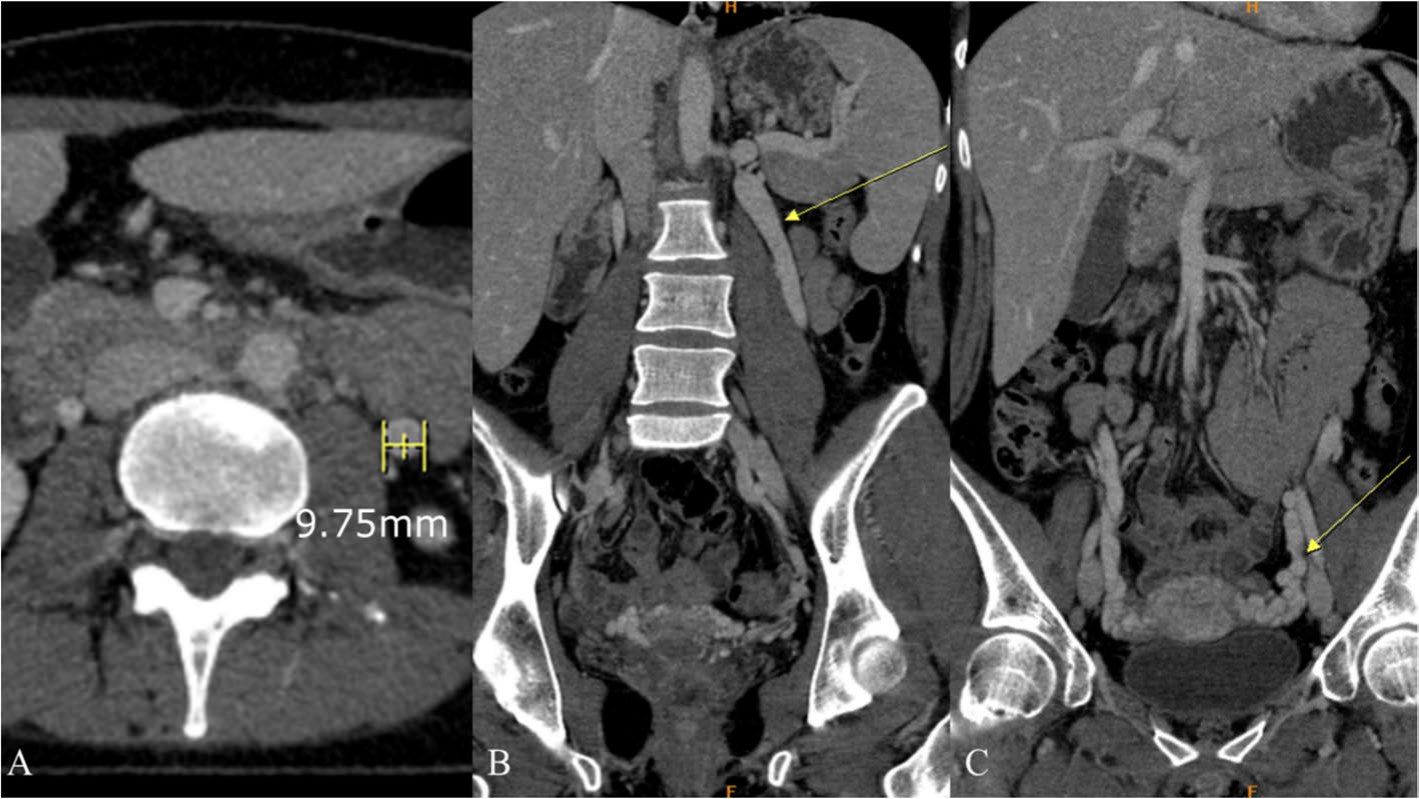
Contrast-enhanced CT examination demonstrating signs of pelvic congestion syndrome distally due to Nutcracker syndrome: **A** axial plane – dilated left ovarian vein (approximately 10 mm in diameter), **B** coronal plane – dilated left ovarian vein (arrow), **C** coronal plane – pelvic varices (arrow)

LRV transposition, the repositioning of the LRV to relieve pressure, is considered the standard of care and provides immediate and long-term relief [7, 8]. Multiple case series indicate that LRV transposition alleviates symptoms in roughly 80% of affected individuals [9, 10]. In persistent symptoms, renal autotransplantation is the subsequent therapy option [9, 11]. Philip et al. [12] conducted a study on renal autotransplantation as a conclusive treatment in 105 patients, reinforcing its efficacy in refractory cases. Transposition of gonadal veins is performed in selected cases to reduce symptoms of pelvic congestion [13, 14] (Fig. 3). Furthermore, new methods are being developed. A recent study from 2020 by a group of authors from China [15] described the successful use of a 3D-printed polyether ether ketone (PEEK) extravascular stent in 28 adolescent patients, illustrating the possibility of personalized solutions for specific cases. More recently, this type of extravascular stent was implanted in an adolescent from Europe, which was published in 2022 [16]. This development highlights the need for a stent design methodology tailored to particular anatomical and pathological conditions.

### Interventional radiology treatment and stent selection

Endovascular stenting is an emerging minimally invasive procedure involving placing a stent inside the LRV to relieve obstruction. Despite positive short-term outcomes, concerns over migration, restenosis, and the need for lifelong anticoagulation limit its widespread adoption [2]. In a study conducted on 75 patients who underwent LRV stenting, stent migration was identified as a significant concern, with a reported incidence of 6.7% [17]. Other published data about stent migration predominantly comprises case reports [18, 19]. Other complications include in-stent restenosis and thrombosis, which occur in about 5% of cases and require careful observation and treatment [2, 3].

Endovascular therapy has exhibited inconsistent success rates. Policha et al. [20] conducted a study including three patients who received endovascular treatment utilizing various stents (a 12 × 14-mm self-expanding nitinol stent, a 14 × 60-mm Wallstent, and a 16 × 40-mm Wallstent), achieving successful outcomes throughout a two-year follow-up period.

Despite its high success rate, LRV transposition sometimes necessitates additional intervention. According to Erben et al. [21], some cases require reintervention, with stenting as a potential option.

Cherfan et al. [22] conducted a trial involving LRV stenting on 18 patients; five previously had LRV transposition but exhibited recurrent complaints. The two-year primary and primary assisted patency rates were 85.2% and 100%, respectively. Larger self-expanding stents of 4–6 cm with 14–16 mm diameters were deemed suitable, while smaller diameters correlated with increased migration rates. The Wallstent (Boston Scientific) has been the predominant stent utilized in these instances; however, novel venous stents are emerging and may achieve broader acceptability.

In recent times, intravascular ultrasound (IVUS) has become increasingly common, enhancing precision during stent placement and reducing complications associated with stent deployment [23, 24].

## Discussion of controversies

Despite the considerable progress in diagnostic methods and treatment options related to NCS, several controversies persist, emphasizing the need for a comprehensive investigation. Nevertheless, the complexity of the syndrome creates challenges for consensus due to the various perspectives on its pathophysiology and management. While progress has been made, the discussion surrounding NCS is still contentious, highlighting the importance of continued scholarly engagement. Therefore, thoroughly investigating these issues is essential to enhance our understanding of NCS and its clinical ramifications.

### Diagnostic challenges: variability in imaging criteria and the need for a consensus algorithm

Diagnostic criteria inconsistency complicates clinical decision-making and leads to inconsistencies in patient care. Doppler ultrasound measurements, including a peak systolic velocity ratio surpassing 4.2 to 5.0, may indicate the existence of NCS; nonetheless, dependence on a solitary diagnostic criterion is inadequate. Consider a scenario where one parameter, for example, the AMA, signifies NCS, while other parameters, like pressure gradient measurements, stay within normal limits. When two of three diagnostic parameters yield positive findings, one may doubt confirming the diagnosis.

Recent evidence indicates that the most precise diagnosis of NCS is attained through an integration of clinical presentation and multimodal imaging results. Meram et al. [25] indicated that catheter-based endovascular evaluation for diagnosing LRV compression syndrome exhibited a significant connection with surgical eligibility and postoperative symptom alleviation. Collateral circulation, over 50% area stenosis observed on IVUS, or a reno-caval pressure gradient of 3 mmHg or greater were strongly associated with a catheter-based endovascular diagnosis of LRV compression syndrome. The lack of a defined scoring system to assess the relative importance of each diagnostic criterion remains an issue. This emphasizes the importance of the next research to create a diagnostic algorithm or scoring system that combines diverse imaging and hemodynamic characteristics, resulting in a more conclusive diagnosis.

### Treatment dilemmas: when to treat pediatric patients and balancing symptomatic relief vs. renal function

The management of NCS in pediatric populations continues to be a topic of active discourse. A considerable number of young individuals exhibit substantial stomach discomfort and pain, despite the left kidney functioning adequately. While certain medical professionals oppose urgent intervention, citing the potential for spontaneous resolution as the child grows, others believe that treatment is necessary to alleviate symptoms and improve overall quality of life. The differences in professional viewpoints highlight a significant absence of agreement on clinical criteria requiring intervention.

This condition raises a crucial question: should the need for symptomatic alleviation take priority over preserving renal function when evaluating treatment necessity? Although conservative care may be appropriate for specific pediatric instances, it is essential to develop a more thorough review approach to determine the necessity of intervention. Factors like the intensity and duration of symptoms and the psychological effects on the patient must be meticulously evaluated in conjunction with anatomical and functional tests.

Moreover, balancing risks and benefits in treatment decisions requires a customized strategy. Multiple aspects, including age, disease severity, anatomical variations, and the preferences of the patient or caregiver, must be considered. For instance, although endovascular stenting may be appropriate for adults with severe symptoms, the potential dangers of migration and restenosis could surpass the advantages in pediatric cases. The integration of IVUS during stent installation signifies a notable enhancement in procedural accuracy by offering real-time visualization of vascular anatomy, ensuring appropriate stent positioning, and enhancing therapeutic results.

### Future directions: 3D-printed stents, novel imaging markers, and long-term studies

**Fig. 4:**
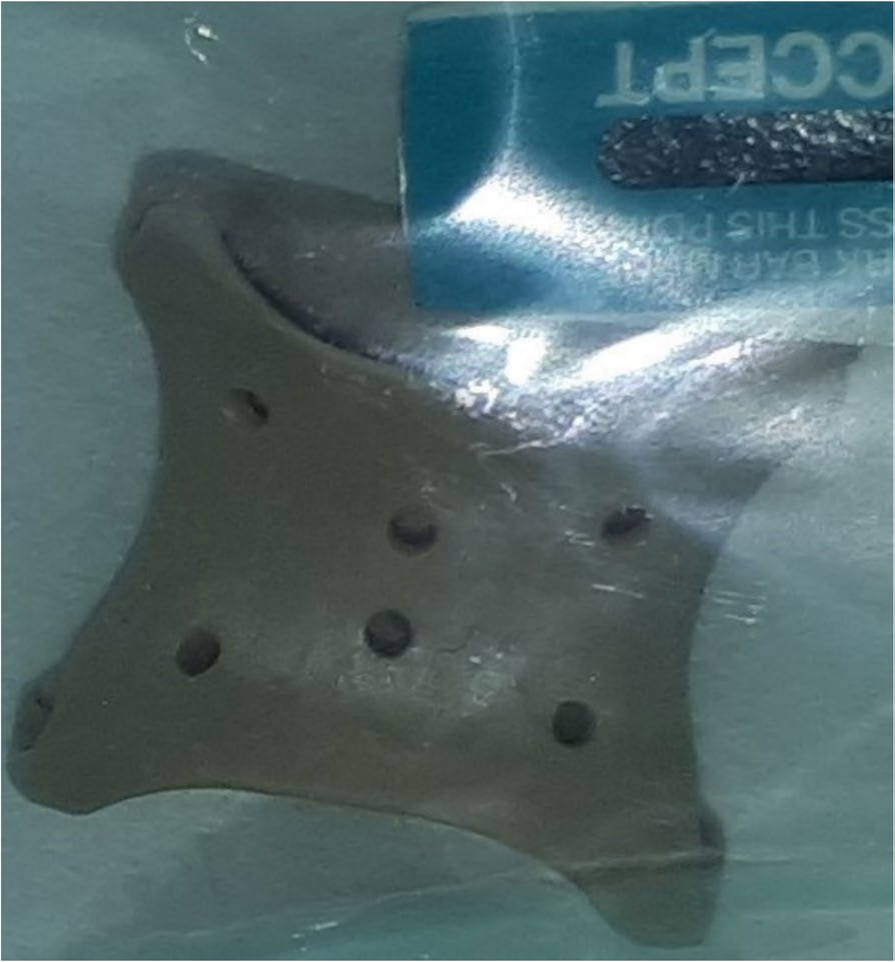
Example of securely packed 3D-printed PEEK extravascular stent (courtesy of Zhang Bo and He Da-li)

Among the novel treatment alternatives, 3D-printed PEEK extravascular stents emerge as a possible substitute for conventional endovascular or surgical procedures. In contrast to endovascular stents, which may present risks, including migration and thrombosis, the extravascular method diminishes the necessity for direct intervention in blood arteries. This stent is constructed from a robust yet biocompatible polymer, providing several significant advantages (Fig. 4).

**Fig. 5:**
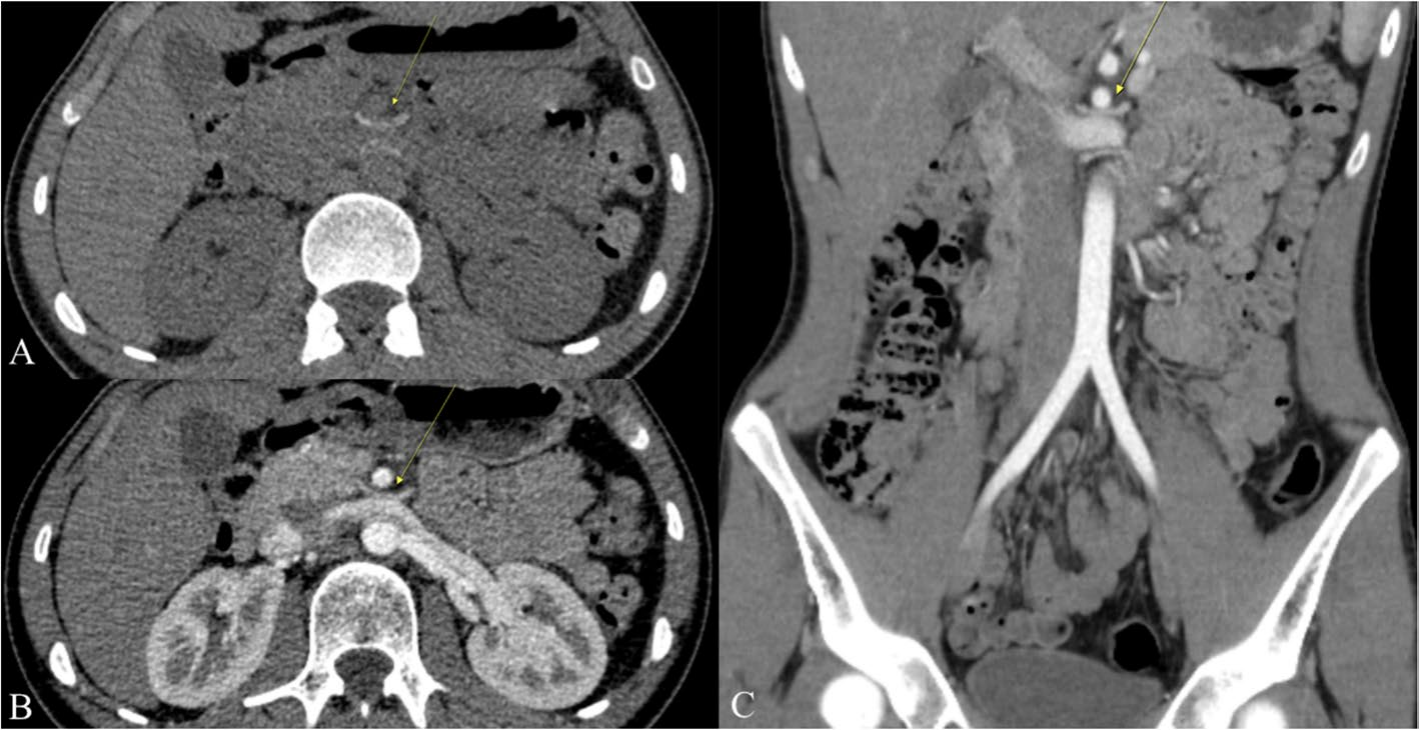
CT examination demonstrating patent 3D-printed PEEK extravascular stent due to Nutcracker syndrome (arrow): **A** non-contrast-enhanced, axial plane, **B** contrast-enhanced, axial plane, **C** contrast-enhanced, coronal plane

A key attribute is its capacity to facilitate expansion. The stent can be extracted or substituted as the youngster develops, mitigating a notable limitation with conventional stents employed in pediatric patients. Laparoscopic stent implantation is a less invasive operation, resulting in reduced recovery periods and a lower risk of problems than open surgical methods (Fig. 5).

**Fig. 6:**
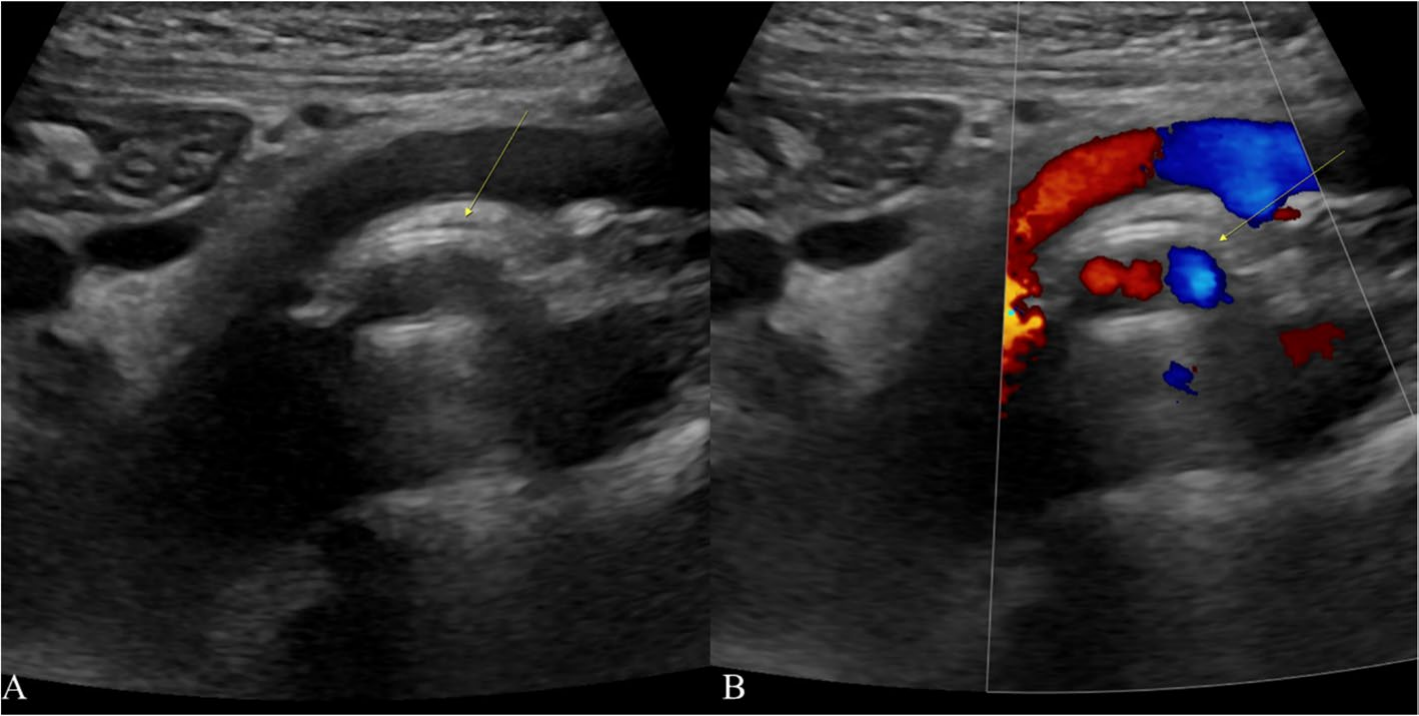
The 3D-printed PEEK extravascular stent in the same patient (arrow): **A** B-mode ultrasound in the sagittal plane, **B** Doppler ultrasound in the sagittal plane

The case of extravascular stent implantation in a European adolescent male, published in 2022 [16], illustrates notable progress in treating Nutcracker syndrome in recent years. Throughout the follow-up period, the patient exhibited no symptoms or problems (Fig. 6). Nonetheless, it is crucial to acknowledge that long-term evidence about the efficacy and safety of 3D-printed stents is still limited.

Future research must resolve persistent issues, including ideal material composition, the likelihood of inflammatory reactions, and the stent's capacity to endure extended mechanical stress. Moreover, the substantial expenses and specialized expertise necessary for 3D printing may restrict availability in resource-limited environments, confounding the evaluation of risks and benefits in treatment choices. Additional studies examining new imaging biomarkers and long-term outcomes of conventional and innovative therapies will be essential in enhancing therapeutic management techniques for NCS.

### Balancing risks and benefits in treatment decisions

The decision-making process regarding conservative, surgical, or minimally invasive interventions requires a careful risk–benefit analysis tailored for each individual. Various factors such as age, symptom severity, anatomical differences, and the preferences of the patient or caregiver must be carefully considered. For example, while endovascular stenting may be suitable for adults with severe symptoms, the associated risks of migration and restenosis might outweigh the benefits in pediatric cases.

Additionally, integrating IVUS during stent placement represents a significant advancement in achieving procedural precision. It provides real-time visualization of vascular anatomy, which helps ensure optimal stent positioning and sufficient luminal expansion. Thus, incorporating IVUS into standard clinical protocols could lead to better patient outcomes and reduce variability in technical success rates.

## Conclusion

Addressing the controversies surrounding the management of NCS requires a multidisciplinary approach that integrates patient-centered care with advancements in diagnostic and therapeutic techniques. Establishing uniform diagnostic criteria and standardized treatment protocols is vital for improving patient outcomes. Additionally, innovations such as 3D-printed stents offer a promising avenue for treatment but need further validation through rigorous clinical trials. The ongoing experiences with pediatric and adolescent cases highlight the importance of personalized care and emphasize the need for continued research to deepen our understanding of this complex vascular disorder.

## Data Availability

Data sharing does not apply to this article as no datasets were generated or analyzed during the current study.
